# Development of novel parameters for pathogen identification in clinical metagenomic next-generation sequencing

**DOI:** 10.3389/fgene.2023.1266990

**Published:** 2023-11-17

**Authors:** Xiwen Jiang, Jinghai Yan, Hao Huang, Lu Ai, Xuegao Yu, Pengqiang Zhong, Yili Chen, Zhikun Liang, Wancen Qiu, Huiying Huang, Wenyan Yan, Yan Liang, Peisong Chen, Ruizhi Wang

**Affiliations:** ^1^ College of Biological Science and Engineering, Fuzhou University, Fuzhou, China; ^2^ Department of Laboratory Medicine, The First Affiliated Hospital, Sun Yat-sen University, Guangzhou, China; ^3^ Guangzhou Darui Biotechnology Co., Ltd., Guangzhou, China

**Keywords:** mNGS, ROC curve, indicators, reads, rank

## Abstract

**Introduction:** Metagenomic next-generation sequencing (mNGS) has emerged as a powerful tool for rapid pathogen identification in clinical practice. However, the parameters used to interpret mNGS data, such as read count, genus rank, and coverage, lack explicit performance evaluation. In this study, the developed indicators as well as novel parameters were assessed for their performance in bacterium detection.

**Methods:** We developed several relevant parameters, including 10M normalized reads, double-discard reads, Genus Rank Ratio, King Genus Rank Ratio, Genus Rank Ratio*Genus Rank, and King Genus Rank Ratio*Genus Rank. These parameters, together with frequently used read indicators including raw reads, reads per million mapped reads (RPM), transcript per kilobase per million mapped reads (TPM), Genus Rank, and coverage were analyzed for their diagnostic efficiency in bronchoalveolar lavage fluid (BALF), a common source for detecting eight bacterium pathogens: *Acinetobacter baumannii*, *Klebsiella pneumoniae*, *Streptococcus pneumoniae*, *Staphylococcus aureus*, *Hemophilus influenzae*, *Stenotrophomonas maltophilia*, *Pseudomonas aeruginosa*, and *Aspergillus fumigatus.*

**Results:** The results demonstrated that these indicators exhibited good diagnostic efficacy for the eight pathogens. The AUC values of all indicators were almost greater than 0.9, and the corresponding sensitivity and specificity values were almost greater than 0.8, excepted coverage. The negative predictive value of all indicators was greater than 0.9. The results showed that the use of double-discarded reads, Genus Rank Ratio*Genus Rank, and King Genus Rank Ratio*Genus Rank exhibited better diagnostic efficiency than that of raw reads, RPM, TPM, and in Genus Rank. These parameters can serve as a reference for interpreting mNGS data of BALF. Moreover, precision filters integrating our novel parameters were built to detect the eight bacterium pathogens in BALF samples through machine learning.

**Summary:** In this study, we developed a set of novel parameters for pathogen identification in clinical mNGS based on reads and ranking. These parameters were found to be more effective in diagnosing pathogens than traditional approaches. The findings provide valuable insights for improving the interpretation of mNGS reports in clinical settings, specifically in BALF analysis.

## Introduction

Next-generation sequencing (NGS) technology, also known as high-throughput or large-scale sequencing technology, can simultaneously and independently sequence thousands to billions of DNA fragments (Boers et al., 2019). Recently, the use of NGS for clinical pathogen diagnosis is widely accepted, with three main applications in the clinical microbiology laboratory: whole-genome sequencing (WGS), metagenomic next-generation sequencing (mNGS), and targeted metagenomics sequencing (tNGS) (Mitchell and Simner, 2019). WGS involves sequencing and assembly of a microbial genome, which is applied to the pure culture growth of a bacterial organism or directly from a viral specimen. WGS is used to identify and epidemiologically track food-borne outbreaks and disease surveillance and to identify multi-drug-resistant nosocomial infections and track the transmission of these organisms. However, this approach is time-consuming due to the microbial culture and has limitations when it is difficult to culture the organism or uncultivable (Brown et al., 2015; Nimmo et al., 2017; Votintseva et al., 2017). The implementation of shotgun and targeted metagenomics sequencing directly from a clinical sample, namely, as mNGS and tNGS, offers the major advantage of eliminating the culture process entirely. The method of mNGS does not rely on traditional microbial culture and can extract all nucleic acids from specimens without bias for high-throughput sequencing (Gu et al., 2019; Han et al., 2019). After biological information analysis, human sequences are removed, and the remaining sequences are compared with pathogen databases to obtain the information on suspected pathogenic microorganisms species (Gu et al., 2019; Han et al., 2019). On the other hand, tNGS involves a selection process before library preparation and sequencing to enrich for the microbial sequences of interest. Enrichment can be achieved using various selection methods such as PCR amplification (commonly known as amplicon sequencing), probe hybridization, and CRISPR–Cas9 utilization (Salipante et al., 2014; Gu et al., 2016). The advantage of tNGS when compared to mNGS approaches is that it overcomes the challenge of amplifying low numbers of microbial sequences within highly cellular samples, often referred to as the “needle in the haystack” dilemma. However, the enrichment process, such as multiplex PCR for specific genes, may introduce target bias (Schlaberg et al., 2017). There has been a growing interest in the use of quasi-metagenomics, which lies between culture-independent metagenomics and pure-culture isolate sequencing. Quasi-metagenomics sequencing involves the analysis of modified microbiomes in food and environmental samples using WGS (Hyeon et al., 2018). In this protocol, the microbiome is modified to concentrate the genomic DNA of a specific food-borne pathogen contaminant, enabling the detection and subtyping of the pathogen in a single workflow (Hyeon et al., 2018).

mNGS can theoretically detect all pathogens in clinical samples and is especially suitable for detecting complex, rare, novel, and atypical infectious diseases (Ge et al., 2021). In particular for some viruses, mNGS might be the only feasible method of detection (Babiker et al., 2020; van Boheemen et al., 2020). mNGS can be used for a variety of common clinical microbiology samples, such as cerebrospinal fluid, whole blood, alveolar lavage fluid, pus, and tissue (Wilson et al., 2014; Doan et al., 2016). In recent years, the successful application of mNGS in clinical cases and studies has gradually increased. The advantage of mNGS in the field of infectious disease diagnosis lies in its ability to detect pathogens that may remain elusive to other conventional detection methods, i.e., the capability to detect difficult-to-culture, rare, or unprecedented pathogenic microorganisms (Wylie et al., 2013; Wilson et al., 2014; Frémond et al., 2015; Doan et al., 2016; Simner et al., 2018).

Current bioinformatics pipelines of mNGS mostly rely on the number of mapped reads for pathogen identification. However, there are significant challenges in interpreting and reporting data from mNGS. Although mNGS is increasingly being applied in clinical settings, there is no guideline as a standard for interpreting mNGS data. Different analysts may have varying interpretations based on their own independent criteria. For the same bacteria being tested, one person may identify it as a pathogenic bacterium and report it as such to the clinician, while another may dismiss it as background detection and provide the clinician with a negative report. When interpreting the mNGS report, it is common practice to classify the detected pathogen as either background or pathogenic bacteria in the specimen based on reads per million mapped reads (RPM), transcript per kilobase per million mapped reads (TPM), genus rank, and coverage. However, the performance of these parameters in pathogen identification has been barely investigated, while it is not yet clear if there are better parameters available. Hence, we developed novel parameters and compared their effectiveness with that of the traditional indexes, such as raw reads, RPM, TPM, and in-Genus rank.

## Materials and methods

### Different indicators of mNGS

In this study, we discussed the diagnostic efficacy of different indicators. According to the results of mNGS, except for the used indicators including raw reads, RPM, TPM coverage, and in Genus Rank, we developed the following novel indicators: 1) read indicators: 10M normalize reads and double-discard reads; 2) rank indicators: Genus Rank Ratio, King Genus Rank Ratio, Genus Rank Ratio*in Genus Rank, and King Genus Rank Ratio*in Genus Rank. The meaning and calculation methods of different indicators are shown in Table 1.

**TABLE 1:** Definitions of the 11 indicators.

Indicator	Annotation
Raw reads	Original read number of the specimen
10M normalize reads	All reads were homogenized with 10M as the standard
Double discard reads	Reads of potentially contaminating microorganisms in the environment and reagents, and reads of target species brought out by similar sequences of other species were excluded
RPM	Reads per million mapped reads
TPM	Transcript per Kilobase per million mapped reads
Coverage	Proportion of genes sequenced in the entire genome
In Genus Rank	Ranking the species of the same genus detected in the sample, ranked from highest to lowest sequence number
Genus Rank Ratio	The genus in which the microbial belongs is ranked from highest to lowest by the number of sequences in all the genera detected in the sample
King Genus Rank Ratio	Genus ranking ratio in different kingdoms
Genus Rank Ratio*in Genus Rank	Joint indicator
King Genus Rank Ratio*in Genus Rank	Joint indicator

bold values means a significant difference.

### Study participants

**FIGURE 1:**
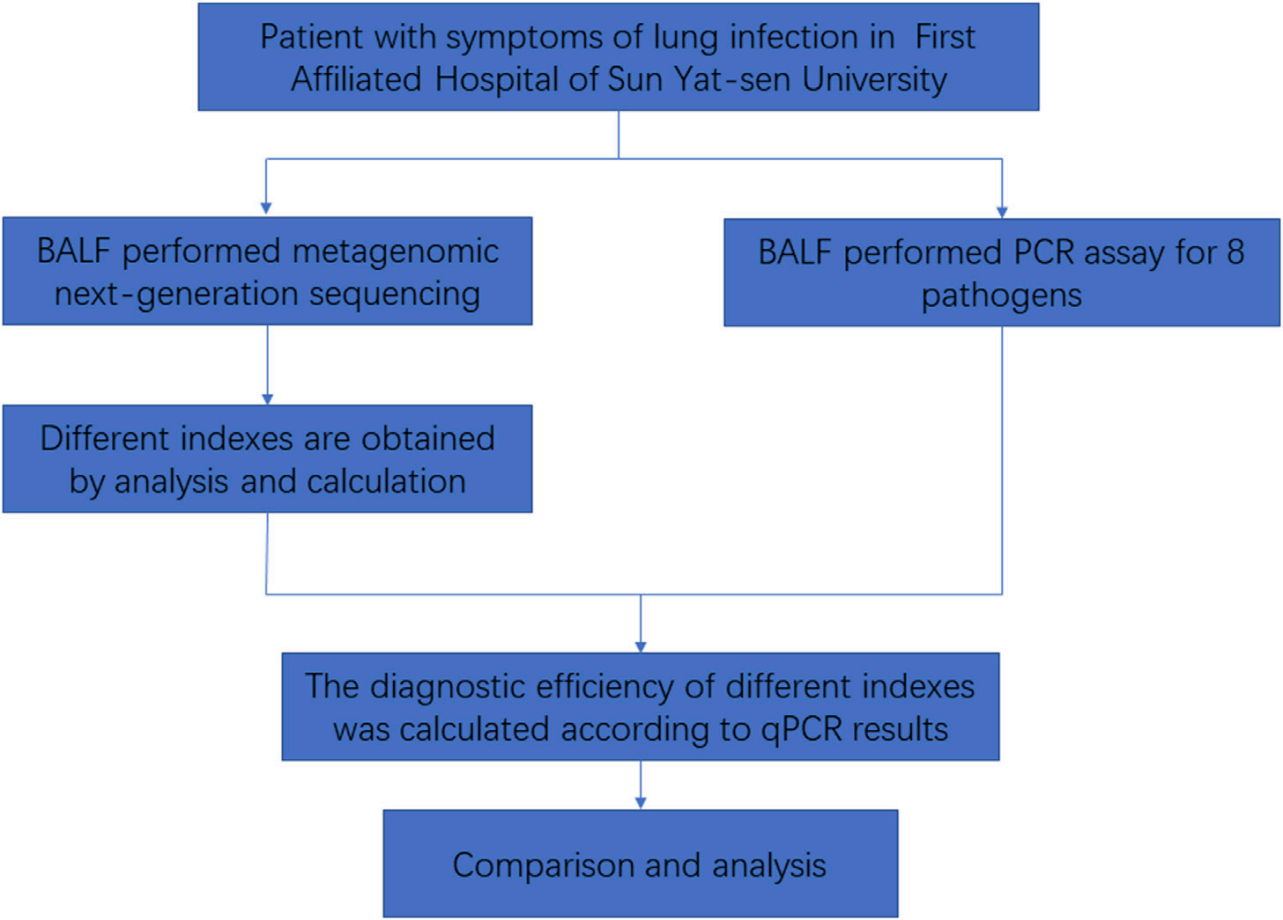
Flowchart of this study.

In this study, we collected bronchoalveolar lavage fluid (BALF) samples from patients at the First Affiliated Hospital of Sun Yat-sen University. These samples will undergo both PCR and mNGS. A total of 605 patients were included in the study. The inclusion criteria were as follows: meeting criteria 1–3 and at least one of criteria 4–8, mentioned as follows: 1) being over 18 years of age; 2) showing pulmonary inflammatory lesions on lung imaging; 3) being willing to participate (either the patient or their guardian); 4) testing positive for the pathogen; 5) experiencing cough, sputum, chest pain, dyspnea, or hemoptysis; 6) presenting with acute fever; 7) showing signs of lung consolidation and/or moist rales; 8) exhibiting increased white blood cell count and C-reactive protein (CRP). Exclusion criteria were as follows: 1) less than 18 years of age; 2) having a clear non-infectious diagnosis; 3) having an insufficient number of specimens; 4) not wanting to participate (either the patient or their guardian). Then, the mNGS results were analyzed to determine the detection status of the following eight pathogens: *Acinetobacter baumannii*, *Klebsiella pneumoniae*, *Streptococcus pneumoniae*, *Staphylococcus aureus*, *Hemophilus influenzae*, *Stenotrophomonas maltophilia*, *Pseudomonas aeruginosa*, and *Aspergillus fumigatus* in each specimen. The data for each index were obtained. The diagnostic efficacy of mNGS was evaluated using the results of PCR as the gold standard (Figure 1).

### Sample processing and DNA extraction from mNGS and PCR

An amount of 400 μl of each patient’s BALF was placed in a 2-ml low-adsorption centrifuge tube, centrifuging at 14,000 g for 3 min. The supernatant was then discarded, and 200 μl of PBS was added to the centrifuge tube for re-suspension. The resulting mixture was incubated with 5% saponins and nuclease at 37°C, 1,000 rpm for 10 min. PBS (1 ml) was added to dilute 15,000 g for 3 min. The supernatant was removed. To the centrifuge tube, 400 μl of PBS was added for re-suspension. The resulting mixture was mixed with lysozyme and glass beads and placed on a vortex mixer’s horizontal platform, stirring intensely at 2,800–3,200 rpm for 30 min. We then used the Micro DNA kit (Guangzhou Darui Biotechnology Co., Ltd., DR-HS-A010, China) to extract DNA following standard protocols. The detected samples included a negative control for detection of cross-contamination and a positive control to monitor DNA extraction efficiency. The DNA concentration was estimated using a Qubit fluorometer (Thermo Fisher Scientific).

### Library preparation and sequencing construction

Based on the principle of high-throughput sequencing, we added a universal sequencing connector to both ends of the extracted DNA fragments, followed by fragment screening to construct the sequencable DNA libraries. We used the TIANSeq DNA library construction kit (TIANGEN, NG104-T3A, China) to construct the DNA libraries, following the established protocols. In order to monitor the accuracy of the experimental steps and filter microbial contaminant sequence, we added equal amounts of the nucleic acid of spike-in control (a plasmid with known amounts constructed by T4 phages) to all the samples. The DNA libraries were quantified using a Qubit fluorometer, and the sizes of the libraries were measured using a Qsep100 (BiOptic Inc.) to all the samples. The constructed mixed libraries were subsequently sequenced on the Ion Torrent platform, ensuring that the resulting qualified data consisted of at least 10 million reads per sample and a Q30 score of 85% or higher. Quality control was maintained by including a negative control sample, which was processed and sequenced in parallel with each sequencing run.

### Bioinformatics analysis and mNGS analysis

After completion of sequencing, the mNGS results were compared and analyzed using the PIP (version: 1.0.0rc4) (DAAN Gene Co.) software in the PIDB_v1.1.1 (DAAN Gene Co.) database. The main steps of the pipeline include trimming of the adapter and low-quality sequences using Fastp (version: 0.21.0), excluding human sequences by mapping to the human reference (GRCh38.p13) using SNAP (version: 1.0.3), aligning the remaining sequence data to microbial databases (PIDB_v1.1.1) consisting of bacteria, fungi, viruses, and parasites by Kraken 2 (version: 2.1.1) and annotating, and then homogenizing the raw taxonomic profile with 10M as the standard.

Following the annotation analysis, double deductions were made for the reads of the detected species to obtain a more realistic taxonomic profile. mNGS experiments with a small amount of nucleic acid input are known to be susceptible to false conclusions due to potential contaminants, especially from molecular biological reagents and the environment. The majority of respiratory samples have a host ratio of over 90%, which means that the effective data ratio is less than 10%. Information on nucleic acid concentration, library concentration, and host ratio can be found in the Supplementary Materials (see Supplementary Table S1). For the first double deduction, we calculated the correlation between the read number of spike-in control added to the negative control sample and in the sample to obtain the read number of the nucleic acid of potential pollutants in the sample. Then, the error is corrected in the sample classification results, which may be due to the introduction of pollutants. Due to the equal amount of spike-in control input in qualified samples and negative control and the positive correlation between the read number of species and the proportion of nucleic acid, the fold change of the spike-in control read number between samples and negative control was equal to the fold change of the contaminated microbe read number between samples and negative control (Zinter et al., 2019). The fold change in the read number of the spike-in control and pollutant in samples and negative control was consistent.

The correlation conversion formula is as follows:

$${Reads}_{spike-in\_sample}/{Reads}_{spike-in\_NC}={Reads}_{\text{pollutants}\_\text{sample}}/{Reads}_{\text{pollutants}\_\text{NC}}.$$

Reads: read number, NC: negative control, and sample: clinical sample.

Due to the homology among species and the extensive contamination of microbial sequences in public databases, the classification of sequences of a single species is often accompanied by the detection of other species, which we consider to be noise data that will interfere with the judgment of results. For the second double deduction, we utilized a multiple linear regression model to quantify the interference relationship between species and then deducted a certain proportion of species read number according to the model. A clinically prevalent species–strain database of BALF was constructed using 1015 BALF samples from the hospital within the last month, which covered 375 species and 1952 strains. The 50-fold reads per strain was simulated using wgsim (version: 1.15-9-g4be6986) and analyzed by PIP to obtain the taxonomic profile. Each target species y may be produced under the joint action of multiple species $x_{k}$ , and the contribution value of each species $x_{k}$ is different, that is, each species $x_{k}$ has a corresponding weight $b_{k}$ .

The variables of the multiple linear regression model between species are composed of species variables:

$$y=b_{0}+b_{1}x_{1}+b_{2}x_{2}+\ldots +b_{k}x_{k}+u_{t}.$$

The weight $b_{k}$ is equal to the fold change of the read number of species y and the reads number of species $x_{k}$ in the simulated data of species $x_{k}$ , that is expressed as follows:

$$b_{k}={Reads}_{y\_x_{simulate}}/{Reads}_{x\_x_{simulate}}.$$

We construct a weighted correlation matrix for the interfering strains of each species. By excluding the noise introduced by species $x_{k}$ , a more realistic read number of species y can be obtained.

After the double deduction, RPM and TPM calculations were performed on the remaining data. Meanwhile, several new pathogen-reporting indexes were introduced by applying feature transformation and feature combination in feature engineering, including “In Genus Rank,” “Genus Rank Ratio,” “King Genus Rank Ratio,” “Genus Rank Ratio*in Genus Rank,” and “King Genus Rank Ratio*in Genus Rank.” To reduce the effects of experimental and human-induced errors in the sample, we take a feature construction approach by implementing the bucket sort algorithm to discretize the read number of species (Koenig and Youn, 2011). Species from the same genus were placed in the same bucket, and immediately after that, the species read number in each bucket was ranked from largest to smallest, and a new index “In Genus Rank” was assigned to each species according to the rank in each bucket. Each bucket was sorted from the largest to the smallest by the read number of the genus. According to the rank percent of the target genus in all genera, a new indicator “Genus Rank Ratio” was obtained. For example, with a total of 100 genera, the read number of the target genus ranks first among all genera, and the “Genus Rank Ratio” is equal to 1 divided by 100, that is, 1%. Similar to the “Genus Rank Ratio,” the index “King Genus Rank Ratio” quantifies the ranking proportion of all genera in the kingdom level to which the target Genus belongs, which is different from the “Genus Rank Ratio.” In feature engineering, new features may better characterize the data by combining several different features, i.e., synthetic features that encode non-linear laws in the feature space by multiplying two or more input features. Here, we combine “In Genus Rank” and “Genus Rank Ratio” as a new feature, named “Genus Rank Ratio*in Genus Rank,” “King Genus Rank Ratio” and “In Genus Rank” were combined, and it is called the “King Genus Rank Ratio*in Genus Rank.”

All reports interpret that indicators were obtained for the eight microorganisms (*A. baumannii*, *K. pneumoniae*, *S. pneumoniae*, *S. aureus*, *H. influenzae*, *S. maltophilia*, *P. aeruginosa*, and *A. fumigatus*) identified in the mNGS results of each specimen.

### PCR assay of the eight pathogens

The PCR assay was performed on the Applied Biosystems™ ProFlex™ PCR system. The primers of the eight pathogens are shown in Table 2. The final reaction volume of 20 µL contained 10 µL of Platinum™ II Taq Hot-Start Green PCR Master Mix (Invitrogen™), 10 μM concentration of each primer (1 mL), and 2 µL of extracted DNA. Thermal cycling conditions were as follows: preheating at 94°C for 2 min, amplification of 40 cycles including denaturation at 94°C for 15 s, annealing at 60°C for 15 s, and extension at 68°C for 15 s. Positive and negative controls were included in each run. PCR products were detected by agarose gel electrophoresis.

**TABLE 2:** Primer sequences of pathogens used for PCR assays.

Pathogen	Forward primer (5′–3′)	Reverse primer (5′–3′)
*A. baumannii*	GCT​CTG​CAA​ATA​GAC​GGC​GAG​ATT​A	TGC​AAC​TGA​GCA​GCC​AAT​TTC​AGA
*K. pneumoniae*	GAT​GCC​AAC​GTG​CCG​CTC​A	CTC​GGC​AGT​ACC​AGA​CAG​CTA​TG
*S. pneumoniae*	TAG​CCG​TTA​CTT​CAT​GTC​CTC​GTT	TCT​GCA​TAA​CTA​ATC​TGG​CTT​ATT​CCT
*S. aureus*	ATA​TGT​GCG​TGT​TTA​TTG​GCA​AAC​GT	GGA​TGA​CGT​AGC​TGA​GCA​AAG​AAA​TGA
*H. influenzae*	AAA​TAC​AAA​CTA​ACG​GCG​AAT​GGA​AC	TAC​CTG​TAG​ATA​ATA​ATC​CAG​CGA​GTG
*S. maltophilia*	GGA​AGC​GGA​CTT​CGG​TCA​G	TAA​GCC​GCT​GAA​TAC​TAA​GGA​TCG
*P. aeruginosa*	GAT​CAA​CAG​CGC​GAA​TAT​CTC​GGA	CGG​ACC​CGC​TGA​TCA​GTC​GAT​ATA
*A. fumigatus*	TCA​AAG​ATA​TCG​ACG​GAT​CGA​CAA	GGA​CAA​TGT​GCA​AGG​CAT​AAG​ATT

### Build machine learning models

Taking all these parameters into consideration, we selected double-discard reads and ranking indicators, such as Genus Rank, Genus Rank Ratio, and King Genus Rank Ratio, for machine learning training. Similarly, the PCR results were used as the standard for comparison. We selected a logistic regression model that randomly splits the data into a 70% training set and a 30% verification set to obtain the calculation formula of the model.

### Statistical analysis

Statistical analysis was carried out by an online statistics tool (http://dxonline.deepwise.com/) and GraphPad prism. The ROC curve, sensitivity, specificity, positive predict value (PPV), and negative predict value (NPV) were calculated using the results of PCR as the reference standard. The significance was fixed at *p <* 0.05.

## Result

### The results of PCR in the eight pathogens

In this study, in order to explore the diagnostic efficacy of different indicators of mNGS, we took the results of PCR as the gold standard for judgment. The result of PCR in the eight pathogens is shown in Table 3.

**TABLE 3:** Identification of the eight pathogens in clinical samples by PCR assay.

	Positive	Negative	Total
*A. baumannii*	32	357	389
*K. pneumoniae*	35	488	523
*S. pneumoniae*	22	447	469
*S. aureus*	21	380	401
*H. influenzae*	29	252	281
*S. maltophilia*	33	357	390
*P. aeruginosa*	33	350	383
*A. fumigatus*	27	525	552

### The assay sensitivity in detecting pathogens

**FIGURE 2:**
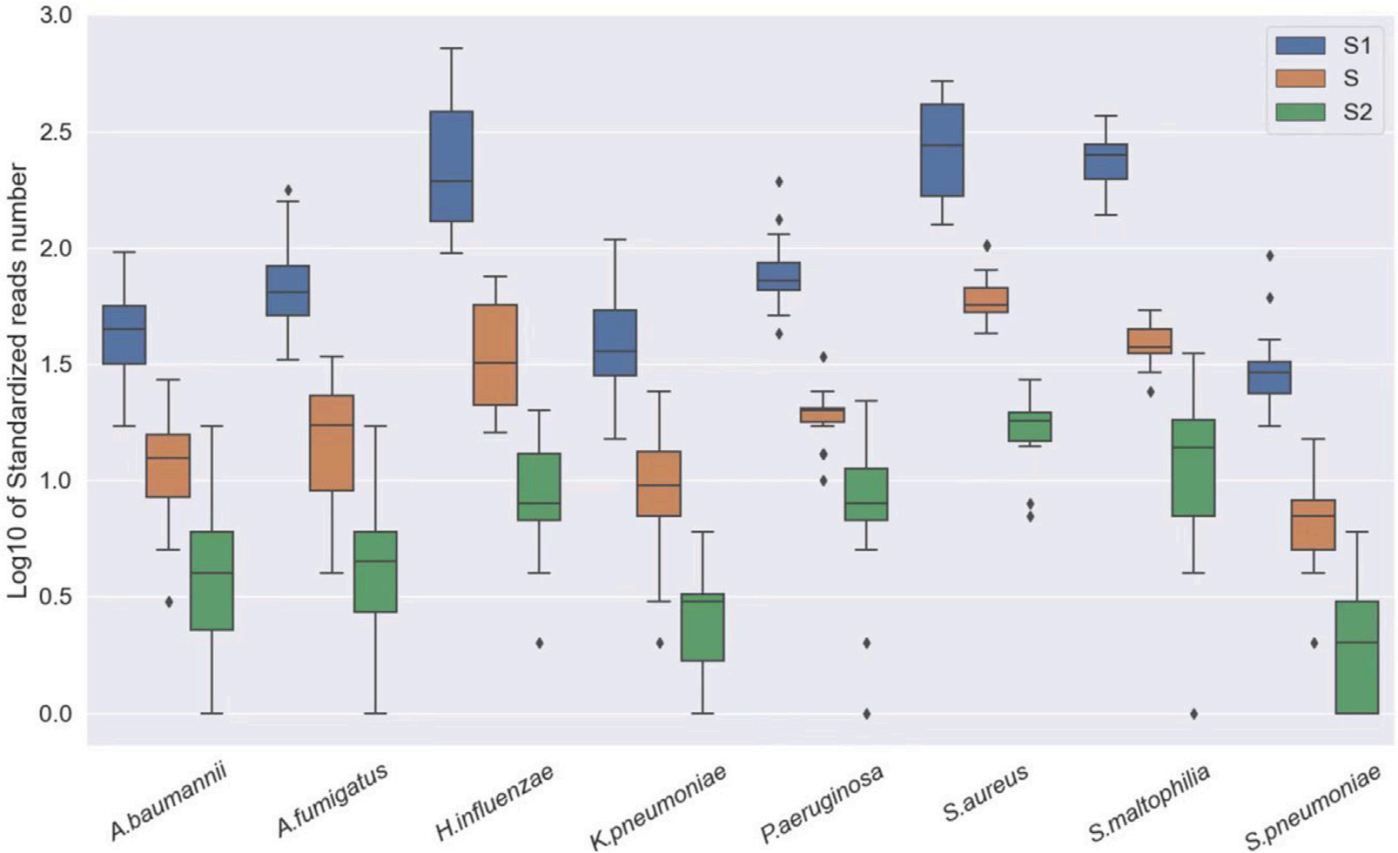
Log10 of standardized read number at different concentrations, S1: *A. baumannii*, *K. pneumoniae*, *H. influenzae*, *S. maltophilia*, and *P. aeruginosa*: 250 CFU/ml; *S. pneumoniae*, *S. aureus*: 500 CFU/ml and *A. fumigatus*: 1,000 CFU/ml. S: *A. baumannii*, *K. pneumoniae*, *H. influenzae*, *S. maltophilia*, and *P. aeruginosa*: 50 CFU/ml; *S. pneumoniae*, *S. aureus*: 100 CFU/ml and *A. fumigatus*: 200 CFU/ml. S2: *A. baumannii*, *K. pneumoniae*, *H. influenzae*, *S. maltophilia*, and *P. aeruginosa*: 100 CFU/ml; *S. pneumoniae*, *S. aureus*: 20 CFU/ml and *A. fumigatus*: 40 CFU/ml.

In clinical specimens, pathogens are often found alongside host cells at varying abundances. Different specimens can have different rates of host cells, which may interfere with the analysis. Therefore, we first conducted a sensitivity analysis of our approach using standard substances. Three pathogen standards in different concentrations were mixed with human DNA (1.25*10^6^ cells/ml) to simulate the host background and were subjected to sequencing. The results showed that even at relatively low concentrations, our approach still demonstrated an excellent performance in detecting microbes (Figure 2).

### The diagnostic efficacy of different indicators in mNGS of the eight pathogens

**FIGURE 3:**
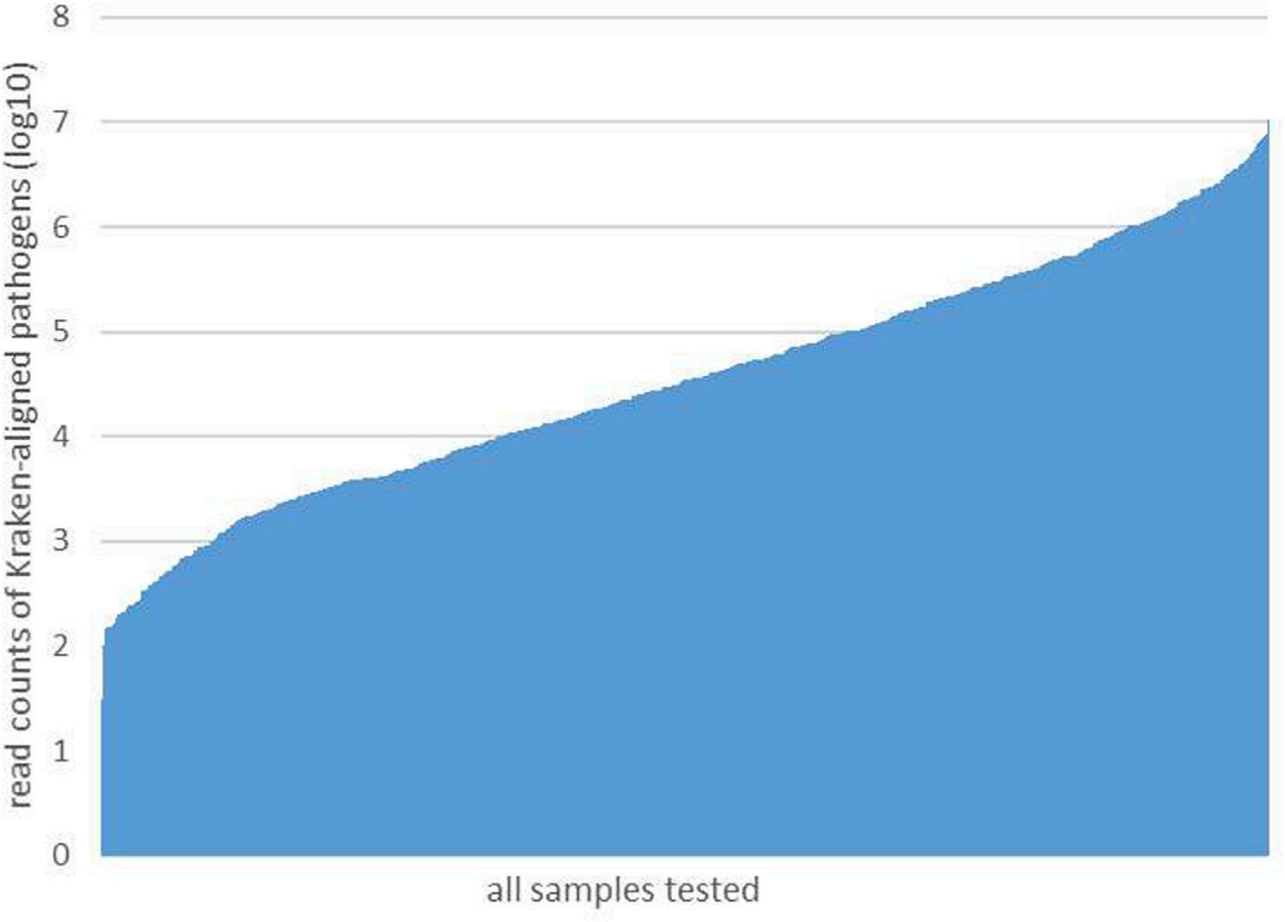
All reads of the detected pathogens in each sample.

**FIGURE 4:**
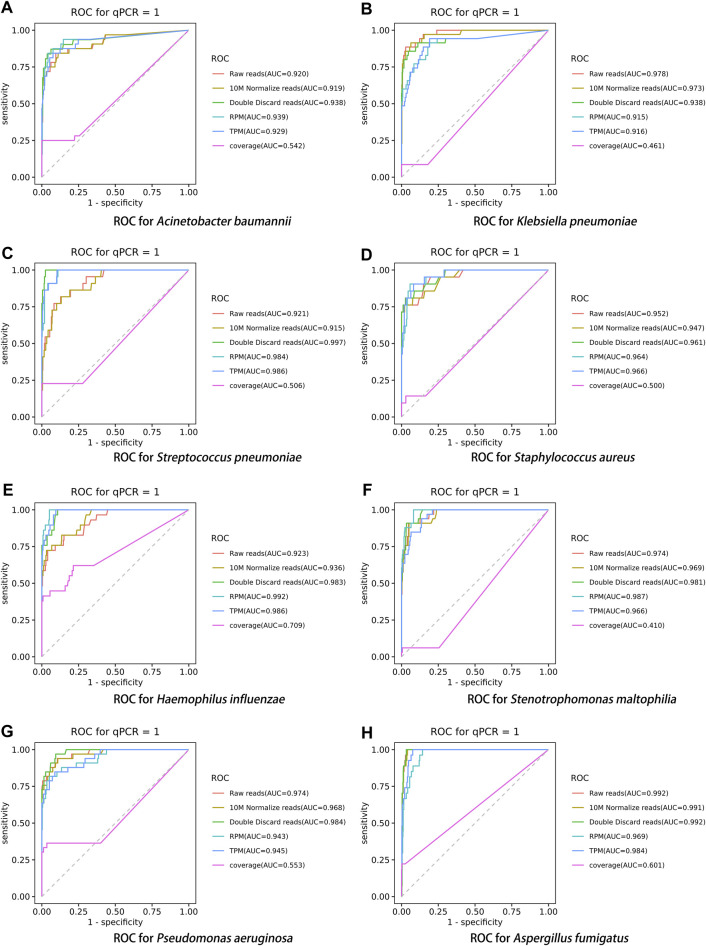
ROC curve of read indicators in the eight pathogens, **(A)** *A. baumannii*, **(B)** *K. pneumoniae*, **(C)** *S. pneumoniae*, **(D)** *S. aureus*, **(E)** *H. influenzae*, **(F)** *S. maltophilia*, **(G)** *P. aeruginosa*, and **(H)** *A. fumigatus*.

**FIGURE 5:**
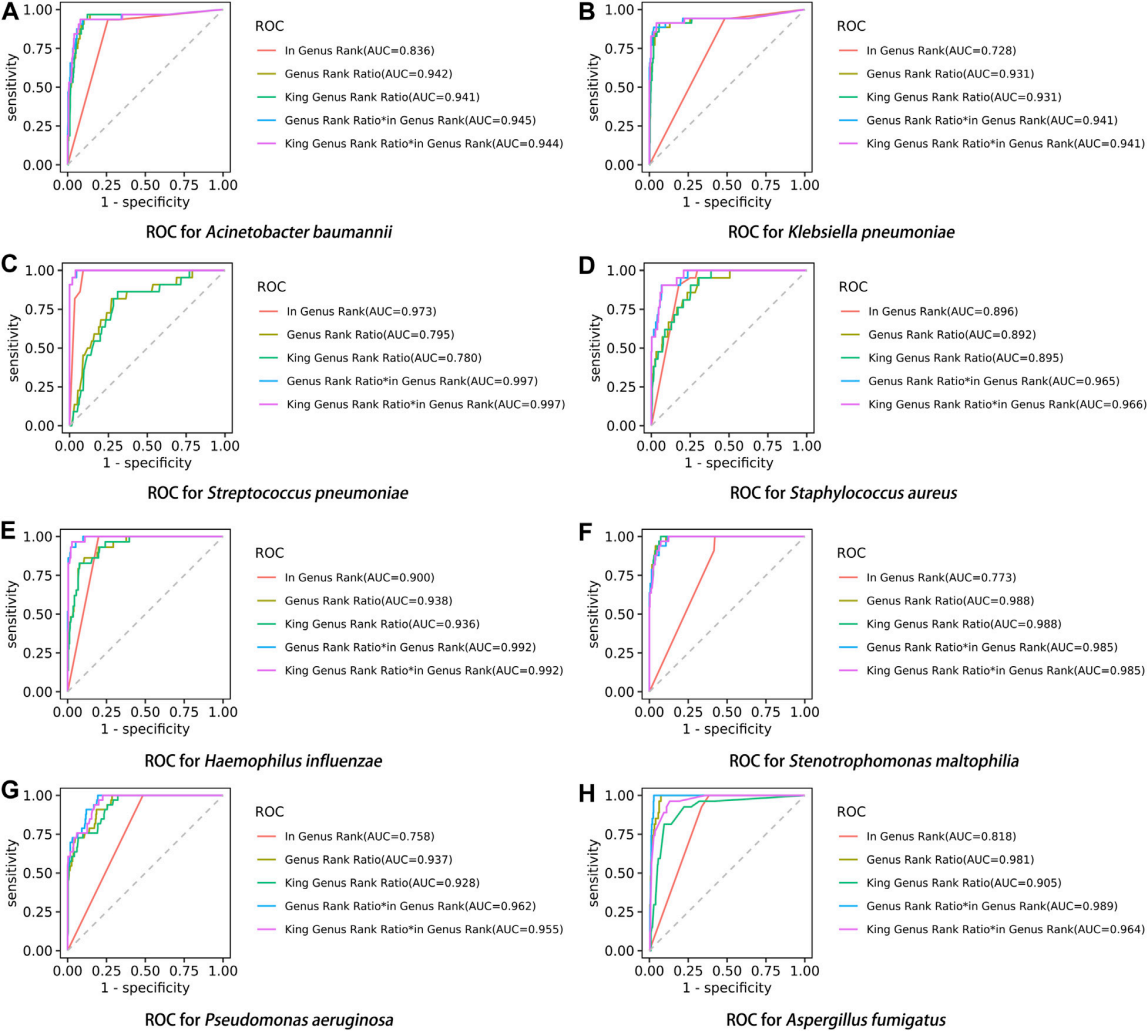
ROC curve of rank indicators in the eight pathogens, **(A)** *A. baumannii*, **(B)** *K. pneumoniae*, **(C)** *S. pneumoniae*, **(D)** *S. aureus*, **(E)** *H. influenzae*, **(F)** *S. maltophilia*, **(G)** *P. aeruginosa*, and **(H)** *A. fumigatus.*

After performing the Kraken 2 analysis on all clinical samples, the total number of mapped pathogen reads ranged from 31 to 10,528,777 (Figure 3; Supplementary Table S2). The ROC curve of read indicators and rank indicators of the eight pathogens using mNGS is shown in Figures 4, 5. The ROC curve enables the determination of the cut-off value for each index, along with its corresponding sensitivity, specificity, PPV, and NPV. The areas under the curve (AUC), cut-off value, sensitivity, specificity, PPV, and NPV of the different indicators in *A. baumannii*, *K. pneumoniae*, *S. pneumoniae*, *S. aureus*, *H. influenzae*, *S. maltophilia*, *P. aeruginosa*, and *A. fumigatus* are listed in Table 4. The results demonstrated that these indicators exhibited good diagnostic efficacy for the eight pathogens. The AUC values of the five read indicators (raw reads, 10M normalized reads, double discard reads, RPM, and TPM) were all greater than 0.9. The corresponding sensitivity and specificity values were also all greater than 0.8, except for the sensitivity of 10M normalized reads in *S. aureus* (0.762) and the sensitivity of raw reads in *H. influenzae* (0.724). Furthermore, the NPV was also higher than 0.95, indicating that these indicators can effectively predict true negative results. Among the five rank indicators, both Genus Rank Ratio*in Genus Rank and King Genus Rank Ratio*in Genus Rank demonstrated better diagnostic efficiency, with AUC values greater than 0.9. The corresponding sensitivity and specificity values were greater than 0.8, except for the specificity of King Genus Rank Ratio*in Genus Rank in *P. aeruginosa* (0.774). Moreover, the NPV was higher than 0.99, highlighting that these two indicators are reliable predictors of true negative results.

**TABLE 4:** Diagnostic efficacy of 11 indicators in mNGS of eight pathogens.

Pathogens	Different index	AUC	Cut-off value	Sensitivity	Specificity	PPV	NPV
*A. baumannii*	Raw reads	0.920	187	0.844	0.905	0.443	0.985
	10M normalize reads	0.919	141	0.844	0.888	0.403	0.984
	Double discard reads	0.938	78	0.871	0.938	0.557	0.988
	RPM	0.939	11326.63	0.844	0.958	0.643	0.986
	TPM	0.929	1312.836	0.844	0.924	0.499	0.985
	Coverage	0.542	16.059	0.25	1	1.000	0.937
	In Genus Rank	0.836	1.5	0.935	0.741	0.244	0.992
	Genus Rank Ratio	0.942	0.236	0.938	0.896	0.447	0.994
	King Genus Rank Ratio	0.941	0.268	0.969	0.873	0.406	0.997
	Genus Rank Ratio*in Genus Rank	0.945	0.273	0.938	0.904	0.467	0.994
	King Genus Rank Ratio*in Genus Rank	0.944	0.267	0.938	0.918	0.506	0.994
*K. pneumoniae*	Raw reads	0.978	296.5	0.886	0.971	0.687	0.992
	10M normalize reads	0.973	120.5	0.914	0.936	0.506	0.993
	Double discard reads	0.938	112.5	0.857	0.953	0.567	0.989
	RPM	0.915	1571.132	0.914	0.809	0.256	0.992
	TPM	0.916	403.752	0.943	0.809	0.262	0.995
	Coverage	0.461	1.016	0.086	1	1.000	0.938
	In Genus Rank	0.728	1.5	0.943	0.514	0.122	0.992
	Genus Rank Ratio	0.931	0.169	0.886	0.953	0.575	0.991
	King Genus Rank Ratio	0.931	0.201	0.914	0.936	0.506	0.993
	Genus Rank Ratio*in Genus Rank	0.941	0.169	0.886	0.971	0.687	0.992
	King Genus Rank Ratio*in Genus Rank	0.941	0.201	0.914	0.955	0.593	0.994
*S. pneumoniae*	Raw reads	0.921	577.5	0.818	0.872	0.239	0.990
	10M normalize reads	0.915	512	0.818	0.868	0.234	0.990
	Double discard reads	0.997	18.5	1.000	0.975	0.663	1.000
	RPM	0.984	10.923	1.000	0.890	0.309	1.000
	TPM	0.986	22.009	1.000	0.897	0.323	1.000
	Coverage	0.506	5.312	0.227	1.000	1.000	0.963
	In Genus Rank	0.973	3.5	1.000	0.913	0.361	1.000
	Genus Rank Ratio	0.795	0.049	0.818	0.729	0.129	0.988
	King Genus Rank Ratio	0.780	0.06	0.864	0.691	0.121	0.990
	Genus Rank Ratio*in Genus Rank	0.997	0.352	1.000	0.955	0.522	1.000
	King Genus Rank Ratio*in Genus Rank	0.997	0.305	1.000	0.962	0.564	1.000
*S. aureus*	Raw reads	0.952	15.5	0.952	0.803	0.211	0.997
	10M normalize reads	0.947	115.5	0.762	0.979	0.667	0.987
	Double discard reads	0.961	31	0.810	0.974	0.633	0.989
	RPM	0.964	2132.809	0.905	0.942	0.463	0.994
	TPM	0.966	806.17	0.905	0.918	0.379	0.994
	Coverage	0.5	0.051	0.143	0.971	0.214	0.953
	In Genus Rank	0.896	1.5	0.905	0.823	0.220	0.994
	Genus Rank Ratio	0.892	0.342	0.952	0.700	0.149	0.996
	King Genus Rank Ratio	0.895	0.314	0.905	0.747	0.165	0.993
	Genus Rank Ratio*in Genus Rank	0.965	0.343	0.905	0.934	0.431	0.994
	King Genus Rank Ratio*in Genus Rank	0.966	0.361	0.905	0.932	0.424	0.994
*H. influenzae*	Raw reads	0.923	324.5	0.724	0.960	0.676	0.968
	10M normalize reads	0.936	97.5	0.828	0.865	0.414	0.978
	Double discard reads	0.983	19.5	1.000	0.892	0.516	1.000
	RPM	0.992	3646.02	1.000	0.948	0.689	1.000
	TPM	0.986	2062.13	1.000	0.905	0.548	1.000
	Coverage	0.709	0.055	0.621	0.786	0.250	0.947
	In Genus Rank	0.900	1.5	1.000	0.801	0.366	1.000
	Genus Rank Ratio	0.938	0.137	0.862	0.893	0.481	0.983
	King Genus Rank Ratio	0.936	0.12	0.828	0.925	0.560	0.979
	Genus Rank Ratio*in Genus Rank	0.992	0.3	0.966	0.948	0.681	0.996
	King Genus Rank Ratio*in Genus Rank	0.992	0.268	0.966	0.972	0.799	0.996
*S. maltophilia*	Raw reads	0.974	175.5	0.909	0.952	0.636	0.991
	10M normalize reads	0.969	139.5	0.909	0.950	0.627	0.991
	Double discard reads	0.981	120	0.909	0.966	0.712	0.991
	RPM	0.987	4996.882	1.000	0.919	0.533	1.000
	TPM	0.966	661.789	0.939	0.863	0.388	0.994
	Coverage	0.41	0.206	0.061	0.994	0.484	0.920
	In Genus Rank	0.773	51	1.000	0.579	0.180	1.000
	Genus Rank Ratio	0.988	0.232	0.970	0.936	0.584	0.997
	King Genus Rank Ratio	0.988	0.274	1.000	0.927	0.559	1.000
	Genus Rank Ratio*in Genus Rank	0.985	0.309	1.000	0.882	0.439	1.000
	King Genus Rank Ratio*in Genus Rank	0.985	0.275	0.970	0.927	0.551	0.997
*P. aeruginosa*	Raw reads	0.974	293.5	0.939	0.894	0.455	0.994
	10M normalize reads	0.968	242.5	0.939	0.891	0.448	0.994
	Double discard reads	0.984	86.5	0.970	0.905	0.490	0.997
	RPM	0.943	24694.8	0.818	0.949	0.602	0.982
	TPM	0.945	3300.692	0.848	0.911	0.473	0.985
	Coverage	0.553	0.084	0.364	0.966	0.502	0.942
	In Genus Rank	0.758	1.5	1.000	0.516	0.163	1.000
	Genus Rank Ratio	0.937	0.159	0.909	0.814	0.315	0.990
	King Genus Rank Ratio	0.928	0.202	0.939	0.743	0.256	0.992
	Genus Rank Ratio*in Genus Rank	0.962	0.224	1.000	0.806	0.327	1.000
	King Genus Rank Ratio*in Genus Rank	0.955	0.269	1.000	0.774	0.294	1.000
*A. fumigatus*	Raw reads	0.992	34.5	1.000	0.964	0.588	1.000
	10M normalize reads	0.991	29.5	1.000	0.960	0.563	1.000
	Double discard reads	0.992	29.5	1.000	0.968	0.616	1.000
	RPM	0.969	566.965	1.000	0.857	0.265	1.000
	TPM	0.984	93.432	1.000	0.924	0.404	1.000
	Coverage	0.601	0.023	0.222	0.998	0.851	0.961
	In Genus Rank	0.818	2.5	1.000	0.616	0.118	1.000
	Genus Rank Ratio	0.981	0.304	1.000	0.926	0.410	1.000
	King Genus Rank Ratio	0.905	0.348	0.815	0.905	0.306	0.990
	Genus Rank Ratio*in Genus Rank	0.989	0.303	1.000	0.971	0.639	1.000
	King Genus Rank Ratio*in Genus Rank	0.964	0.709	0.963	0.869	0.274	0.998

### Pairwise comparisons of different indicators

Using the DeLong test for pairwise comparisons, the AUC of double discard reads was significantly higher than that of raw reads and 10M normalized reads in *S. pneumoniae* and *H. influenzae* (*p <* 0.01). The AUC of double discard reads was significantly higher than that of 10M normalized reads in *S. maltophilia* (*p <* 0.05) (Table 5). The AUC of the double discard reads was greater than 0.9 for all eight pathogens, indicating a high diagnostic efficacy. Compared to RPM and TPM, the AUC of double discard reads was significantly higher in *K. pneumoniae* and *P. aeruginosa* (*p <* 0.05), and compared to RPM, the AUC of double-discarded reads was significantly higher in *S. pneumoniae* (*p <* 0.05) and *A. fumigatus* (*p <* 0.01). By definition, double discard reads further remove host-derived readings from the results, yielding a more accurate representation of pathogen information in the specimen. Therefore, double discard reads could be the preferred option when considering the number of reads in the mNGS report (Table 5).

**TABLE 5:** Pairwise comparisons of different indicators.

		Raw reads–10M normalize reads		Raw reads–double discard reads		10M normalize reads–double discard reads		Double discard–RPM		Double discard–TPM		In Genus Rank–Genus Rank Ratio*in Genus Rank		In Genus Rank–King Genus Rank Ratio*in Genus Rank		Genus Rank Ratio–Genus Rank Ratio*in Genus Rank		Genus Rank Ratio–King Genus Rank Ratio*in Genus Rank		King Genus Rank Ratio–Genus Rank Ratio*in Genus Rank	
*A. baumannii*	AUC	0.92	0.919	0.92	0.938	0.919	0.938	0.938	0.939	0.938	0.929	0.836	0.945	0.836	0.944	0.942	0.945	0.942	0.944	0.941	0.945
	*p*	0.691		0.133		0.12		0.905		0.158		0.000 **		0.000 **		0.731		0.792		0.651	
*K. pneumoniae*	AUC	0.978	0.973	0.978	0.938	0.973	0.938	0.938	0.915	0.938	0.916	0.728	0.941	0.728	0.941	0.931	0.941	0.931	0.941	0.931	0.941
	*p*	0.332		0.19		0.253		0.033 *		0.050 *		0.000 **		0.000 **		0.007 **		0.024 *		0.037 *	
*S. pneumoniae*	AUC	0.921	0.915	0.921	0.997	0.915	0.997	0.997	0.984	0.997	0.986	0.973	0.997	0.973	0.997	0.795	0.997	0.795	0.997	0.78	0.997
	*p*	0.161		0.002 **		0.002 **		0.041 *		0.076		0.001 **		0.001 **		0.000 **		0.000 **		0.000 **	
*S. aureus*	AUC	0.952	0.947	0.952	0.961	0.947	0.961	0.961	0.964	0.961	0.966	0.896	0.965	0.896	0.966	0.892	0.965	0.892	0.966	0.895	0.965
	*p*	0.431		0.566		0.357		0.766		0.593		0.000 **		0.000 **		0.001 **		0.001 **		0.001 **	
*H. influenzae*	AUC	0.923	0.936	0.923	0.983	0.936	0.983	0.983	0.992	0.983	0.986	0.9	0.992	0.9	0.992	0.938	0.992	0.938	0.992	0.936	0.992
	*p*	0.092		0.002 **		0.002 **		0.187		0.695		0.000 **		0.000 **		0.001 **		0.001 **		0.001 **	
*S. maltophilia*	AUC	0.974	0.969	0.974	0.981	0.969	0.981	0.981	0.987	0.981	0.966	0.773	0.985	0.773	0.985	0.988	0.985	0.988	0.985	0.988	0.985
	*p*	0.115		0.092		0.026 *		0.377		0.098		0.000 **		0.000 **		0.273		0.291		0.414	
*P. aeruginosa*	AUC	0.974	0.968	0.974	0.984	0.968	0.984	0.984	0.943	0.984	0.945	0.758	0.962	0.758	0.955	0.937	0.962	0.937	0.955	0.928	0.962
	*p*	0.079		0.108		0.068		0.017 *		0.015 *		0.000 **		0.000 **		0.001 **		0.018 *		0.001 **	
*A. fumigatus*	AUC	0.992	0.991	0.992	0.992	0.991	0.992	0.992	0.969	0.992	0.984	0.818	0.989	0.818	0.964	0.981	0.989	0.981	0.964	0.905	0.989
	*p*	0.53		0.473		0.146		0.003 **		0.052		0.000 **		0.000 **		0.015 *		**0.144**		0.002 **	

Compared to in Genus Rank, the AUC of Genus Rank Ratio*in Genus Rank and King Genus Rank Ratio*in Genus Rank was significantly higher in all the eight pathogens (*p <* 0.01). Compared to the Genus Rank Ratio, the AUC of Genus Rank Ratio*in Genus Rank and King Genus Rank Ratio*in Genus Rank was significantly higher in *K. pneumoniae* (*p <* 0.01 and *p <* 0.05, respectively), *S. pneumoniae* (*p <* 0.01), *S. aureus* (*p <* 0.01), *H. influenzae* (*p <* 0.01), and *P. aeruginosa* (*p <* 0.01). Compared to the Genus Rank Ratio, the AUC of the Genus Rank Ratio*in Genus Rank was significantly higher in *A. fumigatus* (*p <* 0.05) (Table 5). Compared to the King Genus Rank Ratio, the AUC of the Genus Rank Ratio*in Genus Rank and King Genus Rank Ratio*in Genus Rank was significantly higher in *K. pneumoniae* (*p <* 0.05 and *p <* 0.01, respectively), *S. pneumoniae* (*p <* 0.01), *S. aureus* (*p <* 0.01), *H. influenzae* (*p <* 0.01), *P. aeruginosa* (*p <* 0.01), and *A. fumigatus* (*p <* 0.01). The results showed that the two joint indicators (Genus Rank Ratio*in Genus Rank and King Genus Rank Ratio*in Genus Rank) outperformed the other three individual rank indicators (in Genus Rank, Genus Rank Ratio, and King Genus Rank Ratio), indicating that the two joint indicators can be selected for analysis when considering the genus ranking of pathogens in the analysis of mNGS reports (Table 5).

### Construction of identification algorithms through a machine learning model

**FIGURE 6:**
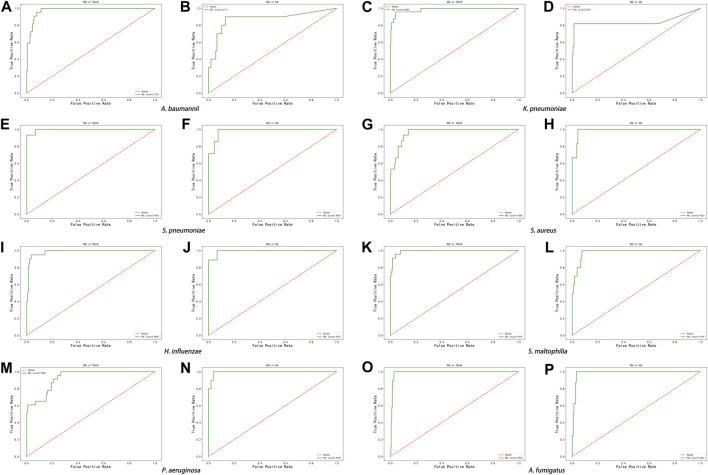
ROC curve of machine learning model in the eight pathogens, **(A,B)** *A. baumannii*, **(C,D)** *K. pneumoniae*, **(E,F)** *S. pneumoniae*, **(G,H)** *S. aureus*, **(I,J)** *H. influenzae*, **(K,L)** *S. maltophilia*, **(M,N)** *P. aeruginosa*, and **(O,P)** *A. fumigatus*.

The score of the logistic regression model is as follows: score = 1/(1 + exp (-logit)). The calculation formula of logit for the training model of each pathogen (Table 6) and the evaluation of the model’s effectiveness (Table 7) were obtained through machine learning. The ROC curves corresponding to each model are shown in Figure 6. The AUC value indicates that the model has a better effect. It is evident that after machine learning training, each model demonstrates improved diagnostic performance.

**TABLE 6:** Calculation formula of the machine learning in the eight pathogens.

Pathogen	Calculation formula
*A. baumannii*	logit = −2.9338 x King Genus Rank Ratio + −0.0867 x In Genus Rank + 1.5019 x Double Discard reads + −3.0728
*K. pneumoniae*	logit = −3.0426 x King Genus Rank Ratio + 1.1003 x Double Discard reads + −1.0055 x In Genus Rank + −3.9922
*S. pneumoniae*	logit = −1.9131 x In Genus Rank + 1.6708 x Double Discard reads + −1.5241 x Genus Rank Ratio + −3.3444
*S. aureus*	logit = −1.9353 x In Genus Rank + 0.8833 x Double Discard reads + −1.9196 x King Genus Rank Ratio + −3.1245
*H. influenzae*	logit = −1.8315 x In Genus Rank + −2.3154 x King Genus Rank Ratio + 0.8692 x Double Discard reads + −3.1204
*S. maltophilia*	logit = −3.6216 x King Genus Rank Ratio + 1.2770 x In Genus Rank + 1.0065 x Double Discard reads + −2.9445
*P. aeruginosa*	logit = 1.6085 x Double Discard reads + −2.4081 x King Genus Rank Ratio + −1.3008 x In Genus Rank + −2.4765
*A. fumigatus*	logit = −2.7780 x Genus Rank Ratio + −1.3057 x In Genus Rank + 0.0499 x Double Discard reads + −4.5830

**TABLE 7:** Evaluation of the model’s effectiveness of the eight pathogens.

Pathogen		Total	Positive	AUC	ACC^a^	Precision	Sensitivity	Specificity	PPV	NPV	AUC-95% CI
*A. baumannii*	Train	272	22	0.9776	0.8824	0.4074	1	0.872	0.4074	1	0.98 (0.9611-0.9941)
	Val	117	10	0.8771	0.8632	0.375	0.9	0.8598	0.375	0.9892	0.88 (0.7268-1)
*K. pneumoniae*	Train	366	24	0.9846	0.8852	0.3594	0.9583	0.8801	0.3594	0.9967	0.98 (0.9647-1)
	Val	157	11	0.8437	0.8599	0.3103	0.8182	0.863	0.3103	0.9844	0.84 (0.645-1)
*S. pneumoniae*	Train	328	15	0.9955	0.9177	0.3571	1	0.9137	0.3571	1	1.00 (0.9866-1)
	Val	141	7	0.9829	0.8582	0.2593	1	0.8507	0.2593	1	0.98 (0.958-1)
*S. aureus*	Train	280	15	0.9648	0.8679	0.2885	1	0.8604	0.2885	1	0.96 (0.9385-0.9911)
	Val	121	6	0.987	0.8926	0.3158	1	0.887	0.3158	1	0.99 (0.967-1)
*H. influenzae*	Train	196	20	0.9852	0.8673	0.4318	0.95	0.858	0.4318	0.9934	0.99 (0.9686-1)
	Val	85	9	0.9927	0.8706	0.45	1	0.8553	0.45	1	0.99 (0.9771-1)
*S. maltophilia*	Train	273	23	0.9922	0.9084	0.4792	1	0.9	0.4792	1	0.99 (0.9837-1)
	Val	117	10	0.9794	0.9145	0.5	1	0.9065	0.5	1	0.98 (0.956-1)
*P. aeruginosa*	Train	268	23	0.9283	0.7724	0.2683	0.9565	0.7551	0.2683	0.9946	0.93 (0.8853-0.9713)
	Val	115	10	0.9943	0.8696	0.4	1	0.8571	0.4	1	0.99 (0.9844-1)
*A. fumigatus*	Train	386	19	0.989	0.943	0.4634	1	0.9401	0.4634	1	0.99 (0.9796-0.9983)
	Val	166	8	0.9866	0.9639	0.5714	1	0.962	0.5714	1	0.99 (0.971-1)

^a^ ACC: accuracy, the probability that the algorithm makes a correct judgment on the current dataset.

## Discussion

Identifying the etiology of infectious diseases is critical in their diagnosis. Traditionally, clinicians make differential diagnoses based on patients’ clinical manifestations and then conduct corresponding tests for each pathogen. However, many pathogenic microorganisms are difficult to cultivate *in vitro* and hard to diagnose through clinical symptoms, which restricts the use of traditional microbiology tests. In comparison, mNGS is able to cover a wider range of pathogens, making it an essential tool for diagnosis (Goldberg et al., 2015).

The positive threshold for mNGS is determined based on the number of microbial-specific sequences and their genome coverage (Chinese Society of Laboratory Medicine, 2020). Since viruses rarely survive in the environment, even a small number of specific sequences can be detected as positive (Miller et al., 2019). It is crucial to avoid reporting environmental bacteria, symbiotic bacteria, and conditioned pathogens that are not clinically relevant. Typically, the higher the number of general sequences, the greater the likelihood of pathogenic microorganisms (tens of specific sequences) (Chinese Society of Laboratory Medicine, 2020). For pathogens of significant clinical concern and those that are difficult to detect, such as *Mycobacterium tuberculosis*, *Yersinia pestis*, and *Brucella*, independent interpretation criteria can be adopted (Chinese Society of Laboratory Medicine, 2020). For instance, if one specific sequence is detected, it can be judged as positive (Miao et al., 2018; Fan et al., 2019). Since the parasite genome is relatively complex and similar to the human genome, it should be interpreted strictly after the sequence specificity is confirmed (Infect Inflamm Rep, 2020). If the detected sequence is a new species, the threshold is not limited, but the homology comparison results must be provided.

In addition to specimen collection, pretreatment, and detection processes, the accuracy of mNGS reports can also be influenced during the result analysis stage. Numerous published studies have investigated the detection efficiency of mNGS on clinical specimens. These studies have found that when the sample contains a high content of pathogenic microorganisms, mNGS displays overall detection performance that is similar to that of PCR, with no significant difference between the two methods. However, in cases where the viral load is low, mNGS may yield false negatives with a specificity of only around 20 million specific sequences, which is lower than that of PCR. To enhance the detection sensitivity in such cases, it is necessary to increase the amount of data used for analysis (Yang et al., 2011; Fischer et al., 2015; Thorburn et al., 2015; Graf et al., 2016; Xie et al., 2019). It is critical to note that mNGS results should not be solely relied upon for clinical decision-making, and negative results should be verified with the patient’s clinical exclusion of infection (Schlaberg et al., 2017). In the current practical application, priority is given to analyzing the mNGS report for pathogens and their species ranking. However, the presence of background contamination and host interference can impact the analysis results, introducing a certain level of subjectivity when combined with clinical symptoms. Therefore, there is a need for new indicators with improved sensitivity and specificity to accurately reflect the true results.

At present, interpretation of mNGS results primarily focuses on the reads and genus rank of the detected pathogens. However, as mentioned previously, different types of pathogens have different criteria for judgment. Relying solely on these two indicators may lead to misinterpretation of the results. Therefore, it is necessary to develop new parameters that can enhance the sensitivity and specificity of these indicators, thereby improving the accuracy of mNGS results. In this study, we developed several new indicators for the diagnosis of mNGS, including 10M normalized reads, double discard reads, TPM, Genus Rank Ratio, King Genus Rank Ratio, Genus Rank Ratio*in Genus Rank, and King Genus Rank Ratio*in Genus Rank. We compared these novel indexes with the existing indicators such as raw reads, RPM, and in Genus Rank. We then analyzed the diagnostic efficacy of these indicators for eight pathogens, namely, *A. baumannii*, *K. pneumoniae*, *S. pneumoniae*, *S. aureus*, *H. influenzae*, *S. maltophilia*, *P. aeruginosa*, and *A. fumigatus*. Based on the analysis of the results for these pathogens, among the five read indicators, double discard reads demonstrated better diagnostic efficiency than the other indicators. It provided a more accurate representation of the actual reads from the pathogens. Among the five ranking indicators, the two combined indicators exhibited superior diagnostic efficiency compared to the three separate indicators. There was no significant difference between the two combined indicators, indicating that either of them can be selected for analyzing the species ranking of pathogens. The analysis results highlight that double discard reads showed higher sensitivity and specificity than raw reads. Additionally, double discard reads better reflected the actual number of detected pathogens during the analysis. The Genus Rank Ratio*in Genus Rank and King Genus Rank Ratio*in Genus Rank also demonstrated improved sensitivity and specificity compared to the in Genus Rank, which is a novel index developed in this study. Importantly, the new indexes exhibited enhanced diagnostic efficiency over the original indexes, thereby increasing the reliability of mNGS results.

However, it is important to acknowledge that in some cases, a single index may not fully reflect the true results. In practical applications, a combination of double discard reads and the two joint ranking indicators can be used for analysis to complement each other and improve the accuracy of diagnosis. Furthermore, the results of the machine learning analysis also indicate that double discard reads, the King Genus Rank Ratio, and in Genus Rank demonstrate good diagnostic performance after training the model.

As high-throughput technology continues to advance, third-generation sequencing technology, such as Nanopore, is already being implemented in clinical laboratories. The key advantages of third-generation technology include long reads (≥500 bp), low capital cost, and short turnaround time (Petersen et al., 2019). Third-generation sequencing has been utilized to bridge the gaps in unfinished genomes sequenced on short-read platforms, thanks to its ability to generate long reads (Bouchez et al., 2018). However, it is important to note that our results were derived from the utilization of mNGS technology and are specifically applicable to mNGS. Different research platforms may have distinct analysis parameters, and it is necessary to conduct further investigations to determine the compatibility of our developed parameters with third-generation sequencing platforms. Additionally, our findings are based on BALF specimens obtained from clinical patients, and additional research is required to evaluate the feasibility of applying our approach to other types of specimens, such as blood, cerebrospinal fluid, and various other body fluids.

## Conclusion

mNGS is a novel technology currently being developed for clinical applications. While it has the potential to identify rare pathogens more quickly than traditional biological detection methods, it still requires improvements to enhance its clinical utility. Therefore, accurate analysis of the mNGS results is crucial. In this study, we analyzed the diagnostic efficiency of several novel indicators. We recommend selecting double discarded reads when considering pathogen reads in the report analysis. For genus ranking, we suggest selecting the two novel indicators: Genus Rank Ratio*in Genus Rank and King Genus Rank Ratio*in Genus Rank. In practical application, when analyzing the mNGS report, using these new indicators in combination can enhance the accuracy of the report, thereby promoting the clinical application of this technology. This will enable the precise detection of pathogens in patients and facilitate timely symptomatic treatment.

## Data Availability

The raw data supporting the conclusion of this article will be made available by the authors, without undue reservation.

## Supplementary material

Supplementary Table S1 Nucleic acid concentration, library concentration and host ratio of all samples.

Supplementary Table S2 Total number of mapped pathogen reads of all samples.
